# Clinical Outcomes of Later‐Generation EGFR‐TKIs for Uncommon EGFR Mutations in NSCLC: A Multicenter Real‐World Study

**DOI:** 10.1111/1759-7714.70179

**Published:** 2025-10-26

**Authors:** Lisa Shigematsu, Tetsuo Tani, Shinnosuke Ikemura, Keiko Ohgino, Kohei Horiuchi, Taro Shinozaki, Shigenari Nukaga, Hideki Terai, Takashi Sato, Katsuhiko Naoki, Koichi Sayama, Yoshitaka Oyamada, Fumio Sakamaki, Kenzo Soejima, Hiroyuki Yasuda, Koichi Fukunaga, Keio Lung Oncology Group (KLOG)

**Affiliations:** ^1^ Division of Pulmonary Medicine, Department of Medicine Keio University School of Medicine Tokyo Japan; ^2^ Department of Respiratory Medicine Graduate School of Medicine University of Yamanashi Chuo Japan; ^3^ Division of Pulmonary Medicine Kawasaki Municipal Hospital Kawasaki Japan; ^4^ Department of Geriatrics and Palliative Medicine Icahn School of Medicine at Mount Sinai New York New York USA; ^5^ Department of Respiratory Medicine, National Hospital Organization Tokyo Medical Center Tokyo Japan; ^6^ Cancer Center, School of Medicine Keio University Tokyo Japan; ^7^ Department of Respiratory Medicine Kitasato University School of Medicine Sagamihara Japan; ^8^ Division of Pulmonary Medicine, Department of Internal Medicine, Tokai University Hachioji Hospital Tokai University School of Medicine Tokyo Japan

**Keywords:** afatinib, NSCLC, osimertinib, real‐world data, uncommon EGFR mutations

## Abstract

**Background:**

Uncommon EGFR mutations, including G719X, L861Q, S768I, and compound mutations, present therapeutic challenges due to limited prospective evidence and variable drug sensitivity. Although later‐generation (i.e., second‐ and third‐) EGFR‐TKIs have shown benefit in some subtypes, real‐world data is limited.

**Methods:**

We retrospectively analyzed patients with advanced or recurrent NSCLC harboring uncommon EGFR mutations diagnosed between 2014 and 2019 at Keio University Hospital and affiliated hospitals. Clinical data were updated through May 2023. EGFR mutations were detected using commercial assays. Common mutations and exon 20 insertions were excluded unless coexisting as compound mutations. Survival outcomes were estimated using the Kaplan–Meier method and compared by log‐rank test; hazard ratios were calculated using the Cox proportional hazards model. Swimmer plots depicted treatment duration by subtype and EGFR‐TKI agents.

**Results:**

Among 35 patients, G719X was the most frequently detected mutation, followed by L861Q and S768I. In addition to these single mutations, various compound mutations involving combinations of G719X, L861Q, S768I, and other rare variants were also observed. While first‐generation EGFR‐TKIs were frequently used initially, 71% of patients eventually received a later‐generation EGFR‐TKI. These patients had significantly longer OS (47.7 vs. 15.5 months; *p* = 0.0177). Multivariate analysis identified non‐use of later‐generation EGFR‐TKIs, liver metastases, and poor performance status as independent poor prognostic factors. Afatinib showed favorable treatment duration in G719X and compound mutations.

**Conclusions:**

Later‐generation EGFR‐TKIs were associated with improved outcomes in patients with uncommon EGFR mutations, with afatinib showing favorable treatment duration in G719X and compound subtypes.

## Graphical abstract

In this multicenter real‐world study, the use of later‐generation EGFR‐TKIs improved clinical outcomes, including overall survival, in patients with uncommon EGFR mutations.

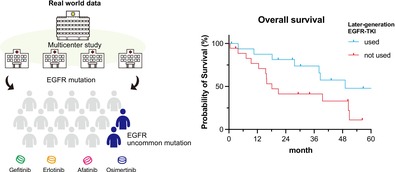

## 1 Introduction

Epidermal growth factor receptor (EGFR) mutations are among the most common oncogenic drivers in non‐small cell lung cancer (NSCLC) [1], particularly in Asian populations and non‐smokers [2]. Classical activating mutations, such as exon 19 deletions and L858R substitutions, which are also referred to as common mutations, account for approximately 80%–90% of all EGFR mutations [3, 4] and are well‐established predictive biomarkers for EGFR tyrosine kinase inhibitors (EGFR‐TKIs) [3, 4, 5, 6]. The treatment paradigm for these common mutations has evolved significantly over the past decade. The pivotal FLAURA trial demonstrated that the third‐generation TKI osimertinib significantly improved progression‐free and overall survival compared to first‐generation TKIs (gefitinib or erlotinib) in patients with common EGFR mutations [7]. Subsequently, the FLAURA2 trial explored the addition of chemotherapy to osimertinib to further enhance efficacy [8], while the MARIPOSA trial investigated a combination of amivantamab (a bispecific EGFR/MET antibody) plus lazertinib (a third‐generation EGFR‐TKI), reflecting continued efforts to optimize outcomes for this patient population [9]. Collectively, these results have established third‐generation EGFR‐TKIs, either alone or in combination regimens, as the standard of care for patients with common EGFR mutations.

Apart from common mutations, the remaining 10%–20% of EGFR mutations are categorized as uncommon mutations, including major subtypes such as G719X, L861Q, S768I, and various compound mutations [10, 11]. These mutations exhibit diverse biological behaviors and variable sensitivity to different EGFR‐TKIs [12, 13], posing unique therapeutic challenges. Indeed, although some studies have reported the efficacy of first‐generation EGFR‐TKIs [14], overall results have been inconsistent and their clinical benefit remains limited [15]. Moreover, post hoc analyses of clinical trials have demonstrated the efficacy of afatinib for major uncommon mutations [16], and the prospective ACHILLES/TORG1834 trial confirmed its ability to significantly prolong progression‐free survival compared to platinum‐doublet chemotherapy [17]. In addition, third‐generation TKIs such as osimertinib have also been evaluated for uncommon EGFR mutations, with the UNICORN study demonstrating promising activity in patients with major uncommon subtypes [18]. Although some prospective studies have demonstrated the efficacy of second‐ or third‐generation EGFR‐TKIs (hereafter referred to as later‐generation EGFR‐TKIs) compared with chemotherapy, no direct head‐to‐head trials have compared first‐, second‐, and third‐generation EGFR‐TKIs in these rare populations, and available data remain limited [19].

Notably, EGFR exon 20 insertion mutations—although historically classified as uncommon—are biologically and clinically distinct due to their well‐established resistance to approved EGFR‐TKIs [20, 21, 22]. These mutations are typically managed with non‐EGFR‐TKI‐based strategies or novel agents and thus were excluded from the present study.

During the study period, clinical practice was shifting from first‐ to later‐generation EGFR‐TKIs, and treatment patterns for uncommon mutations varied considerably in real‐world settings. Yet, evidence describing long‐term outcomes during this transition has remained limited.

We therefore conducted a retrospective multicenter study within the Keio Lung Oncology Group (KLOG) to capture treatment patterns and long‐term outcomes in NSCLC patients with uncommon EGFR mutations. By leveraging an extended observation period, this study aimed to provide real‐world insight into the effectiveness of later‐generation EGFR‐TKIs, including their potential impact on survival, while taking into account relevant confounding factors and sources of bias.

## 2 Materials and Methods

### 2.1 Study Design and Patient Selection

This was a retrospective, multicenter cohort study including all consecutive patients with advanced or recurrent non‐small cell lung cancer (NSCLC) who underwent EGFR mutation testing between 2014 and 2019 across Keio Lung Oncology Group (KLOG) sites, including Keio University Hospital and its affiliated institutions.

**FIGURE 1:**
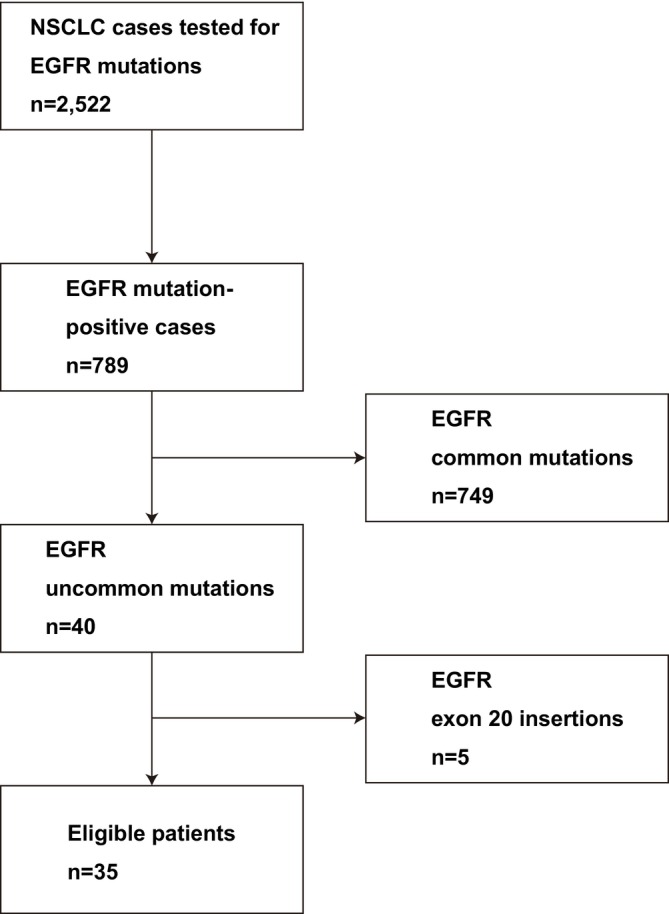
Patient flow diagram. Between January 2014 and December 2019, a total of 2522 tests for EGFR mutation testing were performed at participating institutions. Among them, 789 cases were identified as EGFR mutation positive. After excluding cases with common EGFR mutations (*n* = 749) and exon 20 insertions (*n* = 5), 35 patients with uncommon EGFR mutations were included in the final analysis.

All consecutive eligible patients were included to ensure comprehensive case capture in this rare molecular subset. Patients were eligible if they harbored uncommon EGFR mutations such as G719X, L861Q, S768I, or other rare variants. Classical exon 19 deletions and L858R substitutions were excluded unless present as part of a compound mutation. Exon 20 insertion mutations were also excluded, as these are known to exhibit limited sensitivity to approved EGFR‐TKIs and are generally managed with alternative therapeutic strategies. The patient flow is summarized in Figure 1.

### 2.2 Data Collection

Clinical information was extracted from electronic medical records and included patient demographics (age at diagnosis, sex, smoking status, ECOG performance status), histological subtype, clinical stage, metastatic sites (e.g., brain, liver, pleural effusion), and EGFR mutation subtype. Treatment‐related data such as the line and generation of EGFR‐TKIs used—and survival outcomes—were also collected. All data were updated through May 2023.

EGFR mutation testing was performed using standard, clinically approved assays, including the cobas EGFR Mutation Test, Oncomine Dx Target Test, AmoyDx mutation panels, Cycleave‐PCR, and PNA‐LNA PCR clamp methods.

### 2.3 Outcome Measures

The primary outcomes were time to treatment failure (TTF) and overall survival (OS), both measured from the initiation of EGFR‐TKI treatment. Treatment duration was additionally visualized using swimmer plots, stratified by mutation subtype and EGFR‐TKI agents.

Adverse events were graded according to the common terminology criteria for adverse events (CTCAE), version 5.0. Disease staging was assigned based on the 8th edition of the TNM classification system (UICC/AJCC).

### 2.4 Statistical Analysis

Kaplan–Meier methods were used to estimate TTF and OS, and survival distributions were compared using the log‐rank test. Univariate and multivariate Cox proportional hazards models were applied to identify clinical variables associated with OS. Variables included in the multivariate model were selected based on clinical relevance and univariate significance.

All statistical analyses were performed using GraphPad Prism (version 10, GraphPad Software, San Diego, CA, USA) and JMP (version 18, SAS Institute Inc., Cary, NC, USA), depending on the analysis purpose. Kaplan–Meier curves and log‐rank tests were conducted using Prism, whereas Cox proportional hazards models were performed using JMP. A two‐sided *p* < 0.05 was considered statistically significant.

A 12‐month landmark analysis was also conducted, restricting the survival analysis to patients who survived at least 12 months after EGFR‐TKI initiation to account for potential immortal time bias.

### 2.5 Ethical Considerations

This retrospective study was conducted by the Keio Lung Oncology Group (KLOG) and approved by the institutional review board (IRB) of each participating institution (Keio University IRB number: 20200270). Informed consent was obtained using an opt‐out approach in accordance with local ethical guidelines and institutional policies. The study was conducted in accordance with the Declaration of Helsinki (revised in 2013).

## 3 Results

### 3.1 Clinical Characteristics of the Study Participants

Between January 2014 and December 2019, a total of 2522 tests were performed in patients with advanced or recurrent NSCLC at participating institutions, including repeat tests in some individuals. Among these, 789 cases were positive for EGFR mutations. After excluding those with classical exon 19 deletions and L858R mutations, as well as exon 20 insertions, 35 patients met the inclusion criteria and were included in the final analysis. The screening and selection process is summarized in Figure 1.

Baseline characteristics of the study population are shown in Table 1. Briefly, the median age was 72 years, 49% were male, and 66% were current or former smokers. Most patients (85.7%) had an ECOG PS of 0 to 1. The profile of histological subtype was predominantly adenocarcinoma. Fifteen (40.0%), 4 (11.4%), and 11 (31.4%) patients had brain metastasis, liver metastasis, and malignant pleural effusion, respectively.

**TABLE 1:** Patient characteristic.

Age	*n* (%)
Median (y)	72
≧75	7 (25.0)
Sex	
Men	17 (48.6)
Women	18 (51.4)
Smoking	
Yes	23 (65.7)
No	12 (34.3)
ECOG PS	
0	21 (60.0)
1	9 (25.7)
2	2 (5.7)
3	2 (5.7)
4	1 (2.9)
Stage	
Metastatic	32 (91.4)
Recurrent	3 (8.6)
Site of metastasis	
Brain	14 (40.0)
Liver	4 (11.4)
Pleural	11 (31.4)

**FIGURE 2:**
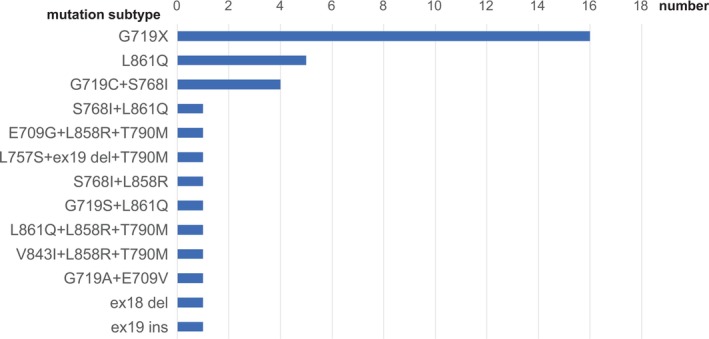
Distribution of uncommon EGFR mutations in the cohort.

Among the 35 patients included in this study, the most frequent uncommon EGFR mutation was G719X alone, observed in 16 patients (45.7%). G719X was also detected as part of compound mutations, including combinations such as G719C with S768I, G719S with L861Q, and G719A with E709V. The second most common mutation was L861Q, identified in 8 patients (22.9%), with 5 having L861Q alone and 3 as part of compound mutations. S768I was detected in 6 patients (17.1%), all in combination with other mutations. Other compound mutations included various complex combinations, such as E709G with L858R and T790M, and L757S with exon 19 deletion and T790M, as well as other patterns shown in Figure 2. Rare mutations such as atypical exon 19 deletion–insertion (*n* = 1, 2.9%) and exon 18 deletion (*n* = 1, 2.9%) were also identified.

### 3.2 Clinical Course

**FIGURE 3:**
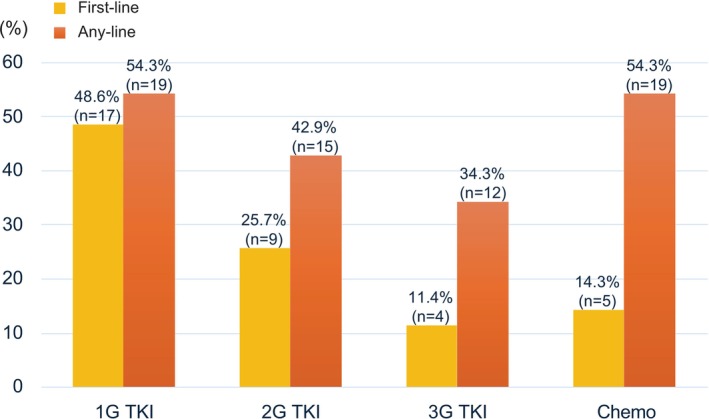
First‐line and any‐line treatment patterns in patients with uncommon EGFR mutations. The yellow bars indicate the proportion of patients receiving each treatment as first‐line therapy, while the orange bars represent the proportion receiving each treatment at any line during their treatment course. Numbers above each bar indicate the percentage and absolute number of patients. 1G TKI, first‐generation EGFR‐TKI (gefitinib or erlotinib); 2G TKI, second‐generation EGFR‐TKI (afatinib); 3G TKI, third‐generation EGFR‐TKI (osimertinib); Chemo, cytotoxic chemotherapy.

Regarding the initial treatment for patients with uncommon EGFR mutations, first‐generation EGFR‐TKIs were the most frequently prescribed, accounting for 48.6% of cases (gefitinib: 22.9%, erlotinib: 25.7%). The second‐generation EGFR‐TKI afatinib was used in 25.7% of patients, while the third‐generation EGFR‐TKI osimertinib was selected as the initial therapy in 11.4% of cases. In addition, cytotoxic chemotherapy was administered as the first‐line treatment in 14.3% of patients (Figure 3). A comprehensive overview of all treatment lines from initial to final therapy for each patient is provided in Figure S1. Although cytotoxic chemotherapy was selected as the initial treatment in some patients, all patients eventually received at least one EGFR‐TKI during their treatment. Examination of treatment use throughout the entire course revealed that 25 patients (71.4%) received at least 1 s‐ or third‐generation EGFR‐TKI, indicating a shift toward later‐generation agents during sequential therapy. First‐generation EGFR‐TKIs remained commonly prescribed, with 19 patients (54.2%) receiving them as any‐line treatment. Specifically, second‐generation EGFR‐TKIs were administered to 15 patients (42.9%), while third‐generation EGFR‐TKIs were used in 12 patients (34.3%). Furthermore, cytotoxic chemotherapy was administered to 19 patients (54.3%) during the treatment period (Figure 3).

### 3.3 Therapeutic Outcomes

This section evaluated both the efficacy and tolerability of EGFR‐TKI treatment in patients with uncommon EGFR mutations, with a primary focus on OS and treatment durability.

#### 3.3.1 Initial EGFR‐TKI Treatment and Time to Treatment Failure

To assess treatment durability, we used time to treatment failure (TTF) as a surrogate marker, as it reflects real‐world treatment persistence.

We analyzed TTF from the initiation of the initial EGFR‐TKI, regardless of treatment line. Patients who received later‐generation EGFR‐TKIs as their initial EGFR‐TKI demonstrated significantly longer TTF compared to those treated with first‐generation EGFR‐TKIs (median TTF: 21.6 vs. 5.2 months; log‐rank *p* = 0.0138; unadjusted HR = 2.234, 95% CI: 1.130–4.415) (Figure S2A).

This analysis included patients who had received cytotoxic chemotherapy prior to EGFR‐TKI initiation, reflecting real‐world treatment sequences.

Given these favorable outcomes in treatment durability, we next evaluated the impact of later‐generation EGFR‐TKI use on overall survival.

#### 3.3.2 Overall Survival Based on EGFR‐TKI Generation

**FIGURE 4:**
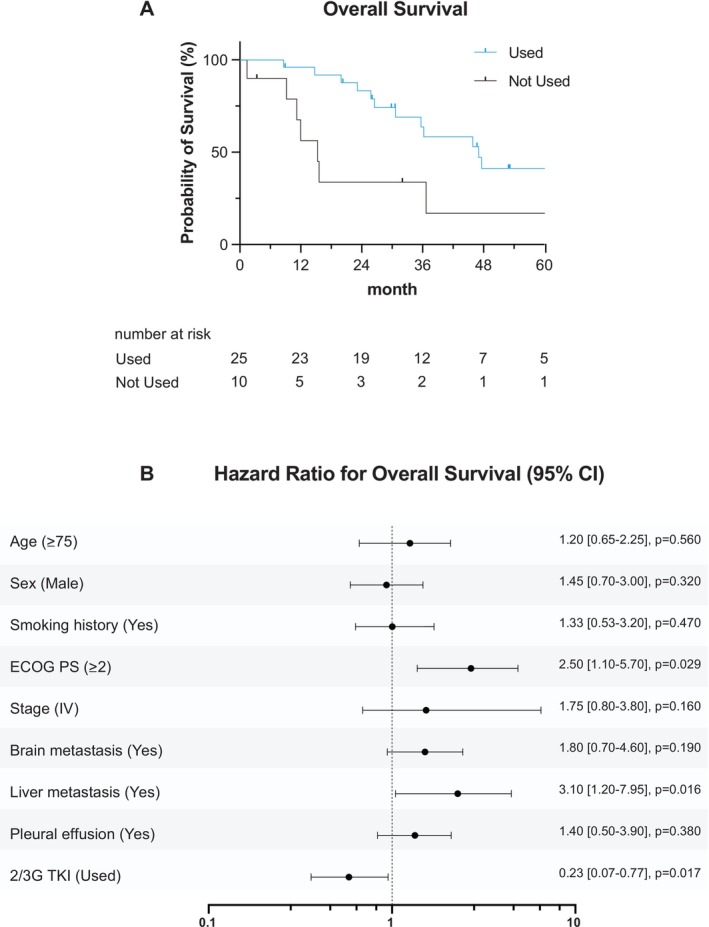
Overall survival according to the use of later‐generation EGFR‐TKIs. (A) Kaplan–Meier curves for overall survival in patients who received later‐generation EGFR‐TKIs (second‐ or third‐generation agents) at any time during their treatment course versus those who did not. The median OS was significantly longer in the later‐generation group (47.7 months) compared to the non‐use group (15.5 months; log‐rank *p* = 0.0177). (B) Forest plot illustrating the results of multivariable Cox proportional hazards analysis for overall survival. Use of later‐generation EGFR‐TKIs remained an independent favorable prognostic factor (hazard ratio [HR], 0.229; 95% confidence interval [CI], 0.068–0.775; *p* = 0.018), after adjusting for relevant clinical covariates. Detailed results of the multivariable model are shown in Table 2.

We first analyzed OS according to the use of later‐generation EGFR‐TKIs. Kaplan–Meier analysis demonstrated significantly longer survival in patients who received later‐generation EGFR‐TKIs (median OS: 47.7 vs. 15.5 months; log‐rank *p* = 0.0177). The unadjusted hazard ratio estimated by a Cox model indicated a more than four‐fold higher risk of death in patients who never received later‐generation EGFR‐TKIs (HR = 4.36; 95% CI: 1.29–14.7) (Figure 4A). In the non‐use group, 6 of 10 patients (60%) harbored G719X mutations.

### 3.4 Univariate and Multivariate Analysis

Univariate Cox regression further revealed that poor ECOG performance status, liver metastasis, and non‐use of later‐generation EGFR‐TKIs were significantly associated with shorter OS (Figure 4B). In the multivariable model adjusting for prespecified covariates, ECOG PS ≥ 2 remained strongly associated with worse OS (HR 2.86, 95% CI 1.36–5.77; *p* = 0.0035), use of later‐generation EGFR‐TKIs was associated with better OS (HR 0.59, 95% CI 0.36–0.99; *p* = 0.0456), whereas liver and brain metastases did not retain statistical significance (Table 2). Notably, brain metastasis was retained in the model a priori because of its clinical relevance, despite only a trend in univariate analysis.

**TABLE 2:** Multivariable cox proportional Hazards analysis for overall survival.

Covariate	Hazard ratio (95% CI)	*p*
ECOG PS ≥ 2	2.86 (1.36–5.77)	0.0035
Liver metastasis (Yes)	1.38 (0.56–3.17)	0.4626
Brain metastasis (Yes)	1.36 (0.76–3.00)	0.2953
2/3G EGFR‐TKI (Used)	0.59 (0.36–0.99)	0.0456

### 3.5 Sensitivity Analyses

To confirm the robustness of our findings and mitigate potential immortal‐time bias, we conducted several sensitivity analyses.

Kaplan–Meier curves were generated from the start of EGFR‐TKI therapy, stratified by the generation of the initial EGFR‐TKI. Patients who received a later‐generation EGFR‐TKI as their initial EGFR‐TKI treatment showed significantly longer overall survival than those who received a first‐generation EGFR‐TKI (median OS: 45.2 vs. 16.7 months; log‐rank *p* = 0.0408; HR = 2.509, 95% CI: 1.034–6.087) (Figure S3A). In a 12‐month landmark analysis limited to patients who survived at least 12 months, the group that eventually received later‐generation EGFR‐TKIs showed numerically longer OS (median OS: 35.6 vs. 24.7 months), although the difference was not statistically significant (log‐rank *p* = 0.1637; HR = 0.527, 95% CI: 0.109–1.456) (Figure S4). While not reaching statistical significance, this finding complements our primary analysis by mitigating immortal time bias.

### 3.6 Treatment Outcomes by EGFR Mutation Subtype

To further evaluate treatment outcomes by mutation subtype, we analyzed swimmer plots depicting treatment duration (as reflected by TTF) during initial EGFR‐TKI treatment for patients with G719X, compound mutations, and L861Q.

**FIGURE 5:**
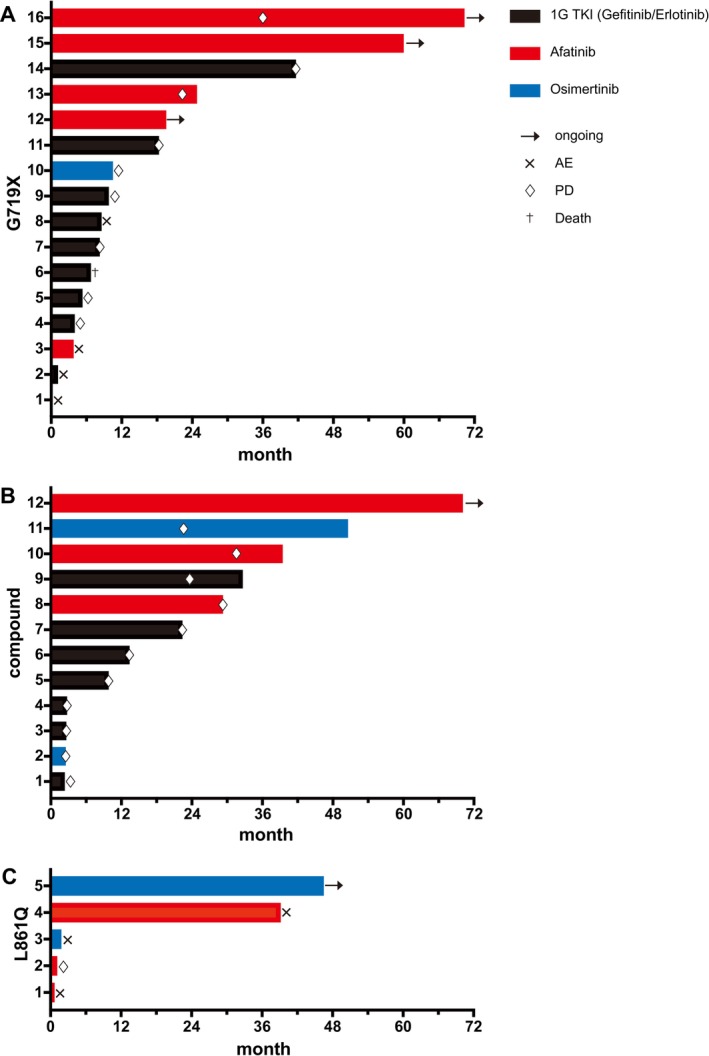
Swimmer plots of TTF stratified by EGFR mutation subtype: G719X, compound mutations, and L861Q, highlighting treatment duration with first‐, second‐, and third‐generation TKIs.

For G719X, the swimmer plot demonstrated that second‐ or third‐generation EGFR‐TKIs achieved longer treatment durations than first‐generation EGFR‐TKIs (Figure 5A). The median TTF for first‐generation EGFR‐TKIs was 7.3 months, compared to 21.7 months for second‐ or third‐generation EGFR‐TKIs (log‐rank *p* = 0.0359) (Figure S5A). Notably, afatinib achieved a TTF exceeding 12 months in 4 out of 5 patients, indicating robust efficacy in this subgroup.

For compound mutations, the swimmer plot revealed a clear benefit for later‐generation EGFR‐TKIs (Figure 5B). The median TTF for first‐generation EGFR‐TKIs was 9.6 months, whereas it was 38.7 months for second‐ or third‐generation EGFR‐TKIs (log‐rank *p* = 0.0356) (Figure S5B). Remarkably, all three patients treated with afatinib achieved a TTF exceeding 24 months, suggesting favorable activity in this challenging subgroup.

For L861Q, only second‐ or third‐generation TKIs were administered, and the median TTF was 1.7 months (Figure S5C). Although the number of cases was limited (*n* = 4), treatment duration was generally shorter than for other subtypes (Figure 5C). Despite the limited sample size, these findings support the use of later‐generation EGFR‐TKIs, particularly afatinib, in selected uncommon EGFR mutations such as G719X and compound types.

### 3.7 Adverse Events

We first summarize adverse events (AEs) during the initial EGFR‐TKI exposure, followed by AEs during subsequent EGFR‐TKI therapy (Table 3). During the initial EGFR‐TKI exposure, 7 of 35 patients (20.0%) discontinued therapy due to AEs (CTCAE ≥ grade 3), most commonly dermatologic toxicity (rash with gefitinib [*n* = 1], rash with erlotinib [*n* = 1], paronychia with afatinib [*n* = 1]), hepatotoxicity (gefitinib [*n* = 1], erlotinib [*n* = 1]), and pneumonitis (afatinib [*n* = 1; grade 4], osimertinib [*n* = 1]). Rash was the most common adverse event across EGFR‐TKIs, particularly with erlotinib and afatinib, consistent with previous reports (Table 3). Hepatotoxicity was observed in 1/8 with gefitinib (12.5%; ≥ 3: 1/8, 12.5%) and 2/10 with erlotinib (20.0%; ≥ 3: 1/10, 10.0%). Afatinib showed diarrhea in 5/12 (41.7%; ≥ 3: 2/12, 16.7%) and paronychia in 2/12 (16.7%; ≥ 3: 2/12, 16.7%). ILD occurred in 1/12 with afatinib (8.3%; ≥ 3: 1/12, 8.3%) and 1/5 with osimertinib (20.0%; ≥ 3: 1/5, 20.0%). Other events with erlotinib included nausea in 2/10 patients (20.0%) and pneumothorax in 1 of 10 (10.0%), while ileus occurred in 1 of 12 patients (8.3%) treated with afatinib.

**TABLE 3:** Incidence of all‐grade and grade ≥ 3 adverse events by EGFR‐TKI.

1st line	Gefitinib	*n* = 8	Erlotinib	*n* = 10	Afatinib	*n* = 12	Osimertinib	*n* = 5
	All Grade	Grade ≥ 3	All Grade	Grade ≥ 3	All Grade	Grade ≥ 3	All Grade	Grade ≥ 3
Rash	2 (25.0)	1 (12.5)	4 (40.0)	2 (20.0)	4 (33.3)	1 (8.3)	1 (20.0)	0 (0.0)
Hepatotoxicity	1 (12.5)	1 (12.5)	2 (20.0)	1 (10.0)	0 (0.0)	0 (0.0)	0 (0.0)	0 (0.0)
ILD	0 (0.0)	0 (0.0)	0 (0.0)	0 (0.0)	1 (8.3)	1 (8.3)	1 (20.0)	1 (20.0)
Diarrhea	0 (0.0)	0 (0.0)	0 (0.0)	0 (0.0)	5 (41.7)	2 (16.7)	0 (0.0)	0 (0.0)
Nausea	0 (0.0)	0 (0.0)	2 (20.0)	0 (0.0)	0 (0.0)	0 (0.0)	0 (0.0)	0 (0.0)
Paronychia	0 (0.0)	0 (0.0)	0 (0.0)	0 (0.0)	2 (16.7)	2 (16.7)	0 (0.0)	0 (0.0)
Hematuria	1 (12.5)	0 (0.0)	0 (0.0)	0 (0.0)	0 (0.0)	0 (0.0)	0 (0.0)	0 (0.0)
Pneumothorax	0 (0.0)	0 (0.0)	1 (10.0)	0 (0.0)	0 (0.0)	0 (0.0)	0 (0.0)	0 (0.0)
ileus	0 (0.0)	0 (0.0)	0 (0.0)	0 (0.0)	1 (8.3)	1 (8.3)	0 (0.0)	0 (0.0)

2nd line‐	Gefitinib	*n* = 2	Erlotinib	*n* = 8	Afatinib	*n* = 4	Osimertinib	*n* = 8
	All Grade	Grade ≥ 3	All Grade	Grade ≥ 3	All Grade	Grade ≥ 3	All Grade	Grade ≥ 3
Rash	0 (0.0)	0 (0.0)	3 (37.5)	2 (25.0)	0 (0.0)	0 (0.0)	1 (12.5)	0 (0.0)
Hepatotoxicity	0 (0.0)	0 (0.0)	1 (12.5)	1 (12.5)	1 (25.0)	0 (0.0)	0 (0.0)	0 (0.0)
Diarrhea	0 (0.0)	0 (0.0)	0 (0.0)	0 (0.0)	1 (25.0)	0 (0.0)	1 (12.5)	0 (0.0)
ILD	0 (0.0)	0 (0.0)	0 (0.0)	0 (0.0)	0 (0.0)	0 (0.0)	1 (12.5)	1 (12.5)
Nausea	0 (0.0)	0 (0.0)	1 (12.5)	0 (0.0)	0 (0.0)	0 (0.0)	0 (0.0)	0 (0.0)
Dizziness	0 (0.0)	0 (0.0)	0 (0.0)	0 (0.0)	1 (25.0)	0 (0.0)	0 (0.0)	0 (0.0)
Pancreatitis	0 (0.0)	0 (0.0)	0 (0.0)	0 (0.0)	1 (25.0)	0 (0.0)	0 (0.0)	0 (0.0)
Paronychia	0 (0.0)	0 (0.0)	0 (0.0)	0 (0.0)	1 (25.0)	0 (0.0)	0 (0.0)	0 (0.0)

Among the 7 patients who discontinued their initial EGFR‐TKI due to AEs, 5 were re‐challenged with EGFR‐TKI therapy, of whom 2 subsequently discontinued because of AEs (rash with erlotinib [*n* = 1], ILD with osimertinib [*n* = 1]).

During subsequent EGFR‐TKI therapy (gefitinib *n* = 2, erlotinib *n* = 8, afatinib *n* = 4, osimertinib *n* = 8), 6 of 15 patients (40.0%) discontinued due to AEs. Rash was seen with 3/8 erlotinib (37.5%; ≥ 3: 2/8, 25.0%) and 1/8 osimertinib (12.5%; ≥ 3: 0/8, 0%); hepatotoxicity with 1/8 erlotinib (12.5%; ≥ 3: 1/8, 12.5%) and 1/4 afatinib (25.0%; ≥ 3: 0/4, 0%); diarrhea with 1/4 afatinib (25.0%; ≥ 3: 0/4, 0%) and 1/8 osimertinib (12.5%; ≥ 3: 0/8, 0%); and ILD with 1/8 osimertinib (12.5%; ≥ 3: 1/8, 12.5%). Afatinib also showed paronychia in 1/4 (25.0%), as well as dizziness 1/4 (25.0%) and pancreatitis 1/4 (25.0%) (all < grade 3).

## 4 Discussion

In this retrospective multicenter cohort, exposure to later‐generation EGFR‐TKIs was associated with improved clinical outcomes, including longer treatment durability and survival, compared with first‐generation agents. These findings are consistent with prior clinical trial evidence and provide real‐world confirmation of the benefit of later‐generation EGFR‐TKIs in patients with uncommon mutations.

Because immortal‐time bias is a concern in retrospective analyses, we performed two complementary approaches. First, OS comparison by initial EGFR‐TKI generation showed a statistically significant difference favoring later‐generation EGFR‐TKIs. Second, in the 12‐month landmark analysis, patients who eventually received later‐generation EGFR‐TKIs showed numerical improvement in OS, although the difference did not reach statistical significance. These results cannot fully eliminate the risk of bias, but the consistent directionality across analyses lends cautious support to the association.

Moreover, we conducted a multivariate Cox regression including ECOG performance status, liver metastasis, and brain metastasis. In multivariable Cox models adjusting for key clinical factors (e.g., performance status, metastatic sites), exposure to later‐generation EGFR‐TKIs remained an independent favorable factor. This supports the clinical relevance of incorporating later‐generation agents into treatment sequences for uncommon EGFR mutations, while acknowledging the need for prospective validation.

Although first‐generation EGFR‐TKIs such as gefitinib and erlotinib have become less commonly used in recent years—especially in countries where later‐generation agents are readily available—they are still prescribed in certain situations, including combination strategies with antiangiogenic agents [23, 24] or in regions with limited drug access [25]. Our cohort spans a transition era in which later‐generation agents became available and includes patients who received chemotherapy prior to EGFR‐TKIs, reflecting real‐world sequencing. The long observation window enabled evaluation of overall survival in this rare population.

Several prior studies have evaluated the efficacy of EGFR‐TKIs in patients with uncommon mutations, including afatinib and osimertinib [11, 13, 16, 17, 18, 26]. For example, the UNICORN trial was a single‐arm study that reported favorable outcomes of osimertinib in treatment‐naïve patients with uncommon EGFR mutations [26]. The ACHILLES study prospectively compared afatinib and chemotherapy in patients with uncommon mutations, highlighting the clinical benefit of afatinib [17]. However, direct comparisons between first‐ and later‐generation EGFR‐TKIs, particularly with respect to overall survival, remain limited in the literature. Our study helps to address this gap using a real‐world population.

The efficacy of EGFR‐TKIs varies by mutation subtype. G719X, L861Q, and compound mutations have shown higher sensitivity to second‐generation TKIs like afatinib in several clinical and preclinical studies [11, 13, 27]. Our results support this finding, as patients with G719X and compound mutations achieved relatively long treatment durations with afatinib. Third‐generation EGFR‐TKIs such as osimertinib and lazertinib have also demonstrated promising activity in patients with some uncommon EGFR mutations [26, 28], though prospective data remain limited. Although both afatinib and osimertinib were used in our cohort, the sample size was insufficient to allow direct comparison between the two agents. Therefore, no conclusions can be drawn regarding their relative efficacy. Notably, the OS difference between the later‐generation and non‐use groups may partly reflect differences in treatment allocation among G719X‐mutant cases. Approximately 60% of patients in the non‐use group harbored G719X mutations but were not treated with second‐generation EGFR‐TKIs. By contrast, in the later‐generation group, a number of G719X patients received afatinib, the only second‐generation agent used in our cohort, and achieved durable disease control. Recent systematic reviews also suggest that second‐generation EGFR‐TKIs may provide greater benefit for G719X mutations compared with first‐generation TKIs and possibly even with osimertinib [19]. This treatment allocation likely contributed, at least in part, to the survival difference observed between the two groups.

Another important aspect is safety. In our cohort, both the overall frequency and the spectrum of adverse events were broadly comparable to those reported in previous studies [29], with dermatologic toxicity, hepatotoxicity, diarrhea, and interstitial lung disease being the most frequently observed events.

Importantly, adverse events were also observed in patients who received EGFR‐TKIs more than once across different treatment lines; however, both their type and frequency were consistent with those described in previous reports [30, 31]. These findings suggest that the safety profile of EGFR‐TKIs in uncommon mutations remains in line with existing evidence, even in patients undergoing re‐treatment.

One strength of this study is the inclusion of patients who received cytotoxic chemotherapy prior to EGFR‐TKI initiation, which reflects real‐world treatment patterns where molecular testing or access to targeted agents may be delayed or evolve based on repeated biopsies or changes in clinical status. By including these patients, we aimed to better represent the practical efficacy of each EGFR‐TKI generation. Another notable strength of our study is the relatively long observation period, which enabled the evaluation of overall survival in this rare patient population. Capturing long‐term outcomes beyond short‐term efficacy measures provides additional clinical relevance and complements previous studies that mainly focused on progression‐free survival.

This study has limitations—including retrospective design, small sample size, residual confounding, and unavoidable risk of immortal‐time bias despite sensitivity analyses—which preclude definitive causal inference. Nevertheless, the convergent signal across methods suggests that later‐generation EGFR‐TKIs may contribute to improved outcomes in uncommon mutations. These results are exploratory in nature and can inform both current clinical sequencing and the design of future prospective studies.

In conclusion, this multicenter real‐world study shows that patients with uncommon EGFR mutations who were exposed to later‐generation EGFR‐TKIs experienced longer treatment durability and overall survival compared with those treated with first‐generation agents. In analyses addressing immortal‐time bias, the initial‐treatment comparison demonstrated a statistically significant survival difference, whereas the 12‐month landmark analysis indicated numerical but non‐significant improvement. Taken together with multivariable modeling, these results provide additional real‐world confirmation of the clinical benefit of later‐generation EGFR‐TKIs in this rare population. Although the retrospective design and limited sample size impose clear limitations, our findings contribute to understanding treatment sequencing in daily practice and reinforce the importance of ensuring access to later‐generation EGFR‐TKIs for patients with uncommon EGFR mutations.

## Author Contributions

L.S. and T.T.: writing – original draft. L.S., T.T., and S.I.: writing – review and editing. L.S., K.H., and S.I.: conceptualization and investigation. L.S., T.T., S.I., K.O., K.H., T.S., S.N., H.T., T.S., K.N., K.S., Y.O., F.S., K.S., H.Y., and K.F.: investigation. T.T., S.I., K.O., S.N., K.N., K.S., H.T., H.Y., and K.F.: supervision.

## Conflicts of Interest

K. N. received research funding from Boehringer‐Ingelheim Japan Inc., Chugai Pharmaceutical Co. Ltd., Ono Pharmaceutical Co. Ltd., Taiho Pharmaceutical Co. Ltd., Abbvie Inc., and Syneos Health, and honoraria for lectures from AstraZeneca K.K. and Chugai Pharmaceutical Co. Ltd. These relationships are outside the submitted work. The other authors declare that they have no known competing financial interests or personal relationships that could have appeared to influence the work reported in this paper.

## Supporting information


**Figure S1:** tca70179‐sup‐0001‐Figures.docx.
**Figure S2:** tca70179‐sup‐0001‐Figures.docx.
**Figure S3:** tca70179‐sup‐0001‐Figures.docx.
**Figure S4:** tca70179‐sup‐0001‐Figures.docx.
**Figure S5:** tca70179‐sup‐0001‐Figures.docx.

## Data Availability

The data that support the findings of this study are available from the corresponding author upon reasonable request.
