# Supplementary material for: Clinical Outcomes of Later‐Generation EGFR‐TKIs for Uncommon EGFR Mutations in NSCLC: A Multicenter Real‐World Study
Source: Thorac Cancer. 2025 Oct 26;16(20):e70179. doi: 10.1111/1759-7714.70179 (PMC12554367; doi:10.1111/1759-7714.70179)
Supplement: Supplementary file 1 — Figure S1: tca70179‐sup‐0001‐Figures.docx. Figure S2: tca70179‐sup‐0001‐Figures.docx. Figure S3: tca70179‐sup‐0001‐Figures.docx. Figure S4: tca70179‐sup‐0001‐Figures.docx. Figure S5: tca70179‐sup‐0001‐Figures.docx. [file TCA-16-e70179-s001.docx]

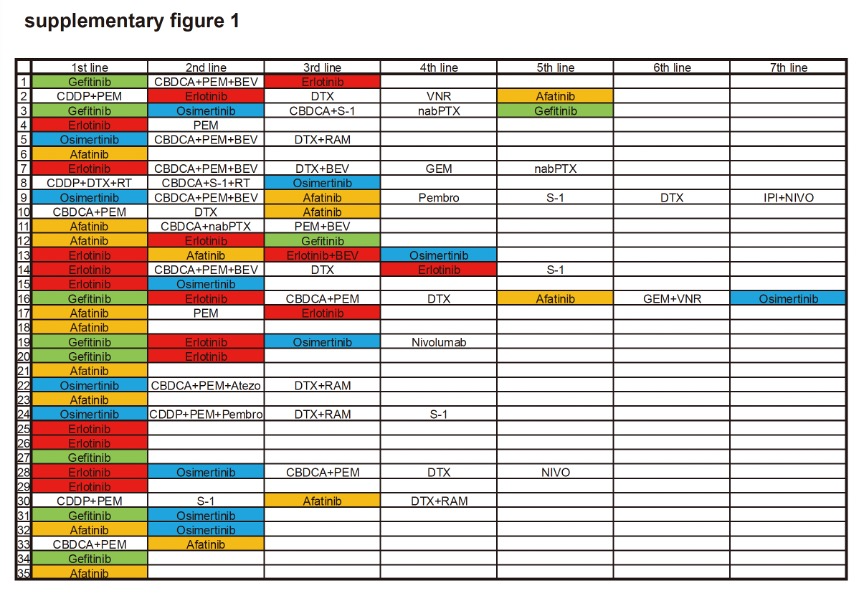


Supplementary Figure 1

**Treatment course from first line to subsequent therapies in all 35 patients. Each row represents an individual patient, with sequential treatment lines shown chronologically from left to right. Colored bars indicate different types of EGFR-TKIs.**

**Treatment regimens are abbreviated as follows: EGFR-TKIs included gefitinib, erlotinib, afatinib, and osimertinib. Chemotherapy agents were abbreviated as CBDCA (carboplatin), CDDP (cisplatin), PEM (pemetrexed), DTX (docetaxel), GEM (gemcitabine), nabPTX (nab-paclitaxel), VNR (vinorelbine), and TS-1 (tegafur–gimeracil–oteracil). Additional agents included RAM (ramucirumab), BEV (bevacizumab), Pembro (pembrolizumab), Atezo (atezolizumab), NIVO (nivolumab), and IPI (ipilimumab). Combination regimens such as CBDCA+PEM+BEV, CBDCA+PEM+Atezo, and CDDP+PEM+Pembro indicate multi-agent therapies, and IPI+NIVO represents the combination of ipilimumab and nivolumab. RT stands for radiotherapy.**

Supplementary Figure 2.

Kaplan–Meier curves of time to treatment failure (TTF) from initiation of the initial EGFR-TKI according to TKI generation. Patients treated with second- or third-generation EGFR-TKIs (2G/3G TKI, blue line) showed significantly longer TTF compared with those who received first-generation EGFR-TKIs (1G TKI, black line). Numbers at risk are shown below the figure.

Supplementary Figure 3.

Kaplan–Meier curves of overall survival (OS) from initiation of the initial EGFR-TKI according to TKI generation. Patients treated with second- or third-generation EGFR-TKIs (2G/3G TKI, blue line) showed significantly longer TTF compared with those who received first-generation EGFR-TKIs (1G TKI, black line). Numbers at risk are shown below the figure.

Supplementary Figure 4.

Kaplan–Meier curves of overall survival (OS) in a 12-month landmark analysis, stratified by the use of second- or third-generation EGFR-TKIs. Numbers at risk are shown below the figure.

Supplementary Figure 5.

Kaplan–Meier curves of time to treatment failure (TTF) stratified by EGFR-TKI generation (1G TKI vs 2G/3G TKI) for each uncommon EGFR mutation subtype: (A) G719X, (B) compound mutations, and (C) L861Q.

1G TKI = first-generation EGFR-TKI (gefitinib or erlotinib); 2G TKI = second-generation EGFR-TKI (afatinib); 3G TKI = third-generation EGFR-TKI (osimertinib).
